# A role for surgery in primary pancreatic B-cell lymphoma: a case report

**DOI:** 10.1186/1752-1947-2-167

**Published:** 2008-05-19

**Authors:** Theodore Liakakos, Evangelos P Misiakos, Dimitrios Tsapralis, Irene Nikolaou, Gabriel Karatzas, Anastasios Macheras

**Affiliations:** 1 Third Department of Surgery, University of Athens School of Medicine, Attikon University Hospital, Rimini 1 Street, Chaidari, Athens, Greece; 2 Department of Pathology, University of Athens School of Medicine, Attikon University Hospital, Rimini 1 Street, Chaidari, Athens, Greece

## Abstract

**Introduction:**

Primary pancreatic lymphoma is a very rare but manageable malignant tumour which may be clinically confused as a pancreatic carcinoma. This case report demonstrates the value of surgical resection in the management of pancreatic lymphoma.

**Case presentation:**

We report a case of a 65-year-old man who was admitted with obstructive jaundice, vague upper abdominal pain and weight loss. Ultrasonography and computed tomography showed a mass at the head of the pancreas which was compressing the bile duct. The patient underwent pancreaticoduodenectomy. Histopathologic and immunohistochemical assessment of the pancreatic lesion established the diagnosis of a diffuse, extranodal, high-grade B-cell non-Hodgkin's lymphoma. Several doses of chemotherapy were administered postoperatively and at present the disease remains in remission.

**Conclusion:**

The favourable outcome for this patient and a thorough review of the literature underline the important role that operative resection may have in the management of at least the early stage of primary pancreatic lymphoma.

## Introduction

Non-Hodgkin's lymphoma (NHL) most frequently arises from the lymphatic system, with the gastrointestinal (GI) tract representing the most commonly involved extranodal site, accounting for half of such cases. In Western series, GI lymphoma occurs principally in the stomach, followed by the small bowel and the colon [1]. Primary involvement of the pancreas in lymphoma is rare, representing a small fraction (less than 1 to 2%) of all pancreatic malignancies [2]. To distinguish primary pancreatic lymphoma (PPL) from secondary involvement of the pancreas by NHL, Behrns' diagnostic criteria include: a predominant pancreatic mass with gross involvement of only the peripancreatic lymph nodes; no hepatic or splenic involvement; no palpable superficial lymphadenopathy; no enlargement of the mediastinal lymph nodes on chest radiograph; and a normal leukocyte count [3].

Current standard management for PPL has relied mainly on the use of various chemotherapeutic protocols, such as the commonly used regimen of cyclophosphamide, doxorubicin, vincristine and dexamethazone (CHOP). The role of surgery in the management of pancreatic lymphoma has been limited to obtaining diagnostic tissue samples or to bypassing biliary obstruction [4]. More recently, less invasive techniques, such as image-guided percutaneous biopsy, have successfully provided a tissue diagnosis in more than 80% of patients [5].

Although initial results with chemotherapy were encouraging, recent studies with longer follow up of patients with NHL treated with standard chemotherapy with or without radiotherapy have demonstrated higher recurrence rates, particularly for patients with abdominal lymphomas [6]. Given the modest results of standard chemotherapy, the known benefits of surgical resection in the prognosis of other GI lymphomas [7], and recent improvements in clinical outcome after radical pancreatic surgery, the role of surgical resection in the treatment of pancreatic NHL is being reassessed [2].

Here we present the case of a patient initially managed as suffering from adenocarcinoma of the pancreas, who ultimately proved to have PPL. The combined treatment of radical surgical resection and chemotherapy resulted in a good clinical outcome.

## Case presentation

A 65-year-old man with a 4-week history of mild epigastric pain radiating to the back (exacerbated after meals), progressive obstructive jaundice, anorexia and weight loss of 3 kg was admitted to our Department of Surgery in November 2005. There was no history of a change in bowel habit, melena, haematochezia, pancreatitis, liver or gallbladder disease. The patient had undergone open repair of an abdominal aortic aneurysm 5 years previously and he was a heavy smoker (40 pack-years) and had a high alcohol intake (300 ml/day).

Upon admission, the patient was icteric, afebrile and had normal vital signs. Physical examination revealed normal bowel sounds, deep jaundice and mild tenderness in the epigastrium and right upper quadrant, without evidence of peripheral lymphadenopathy, palpable mass or hepatosplenomegaly. The patient had an unremarkable haematological profile, but the liver function tests were abnormal: aspartate transaminase (AST) 253 IU/l, alanine transaminase (ALT) 393 IU/l, total bilirubin 7.9 mg/ml, direct bilirubin 6.41 mg/ml, alkaline phosphatase 236 U/l and gamma glutamyl transpeptidase (GGT) 462 U/l. Both serum carcinoembryonic antigen titre and serum carbohydrate antigen titre were normal.

**Figure 1:**
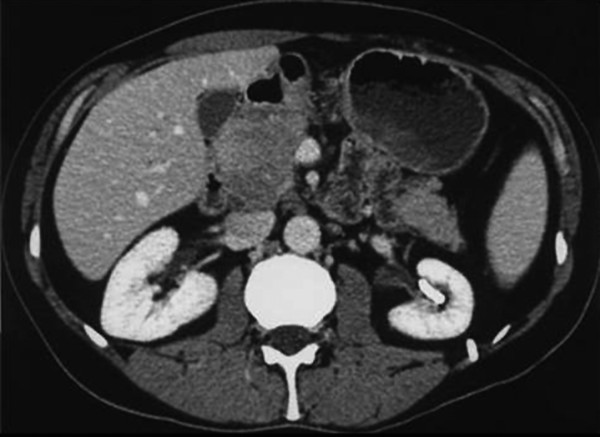
Contrast-enhanced helical CT scan. This scan demonstrates the heterogeneous enhancement of a large tumour of the head of the pancreas, in contact with the superior mesenteric vein (SMV), without signs of infiltration or encasement of the vein, or the superior mesenteric artery (SMA).

The patient underwent ultrasonography of the liver, biliary tract and pancreas, which revealed a hypoechoic mass at the head of the pancreas. Further assessment of this finding with helical, contrast-enhanced abdominal computed tomography (CT) demonstrated the presence of a large, hypodense, non-homogeneous lesion at the head of the pancreas, with a maximal diameter of 5 cm, without dilatation of the main pancreatic duct (Figure 1). Limited lymphadenopathy was also detected in the peripancreatic and the para-aortic regions. The tumour was in contact with the superior mesenteric vein (SMV) without signs of infiltration or encasement of the vein or of the superior mesenteric artery (SMA). The patient also underwent upper GI endoscopy, which revealed mild gastritis in the gastric antrum without evidence of malignancy in the stomach or duodenum. A CT-guided fine needle aspiration (FNA) biopsy was not diagnostic, and the patient was scheduled for exploratory laparotomy.

**Figure 2:**
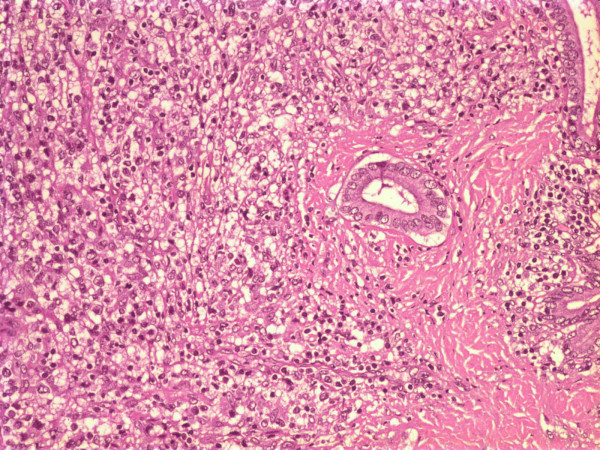
Neoplastic lymphoid population surrounding pancreatic duct with no evidence of lymphoepithelial lesions (H/E × 200).

**Figure 3:**
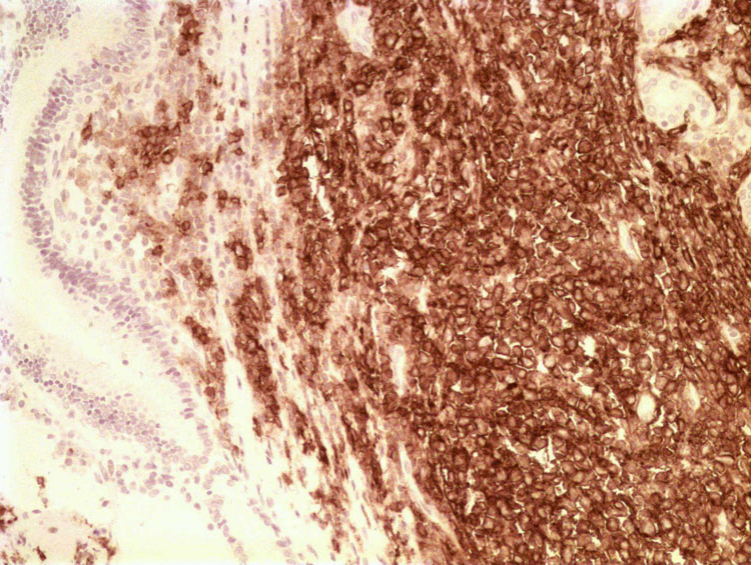
CD20 positivity of neoplastic lymphoid cells (CD20 × 200).

**Figure 4:**
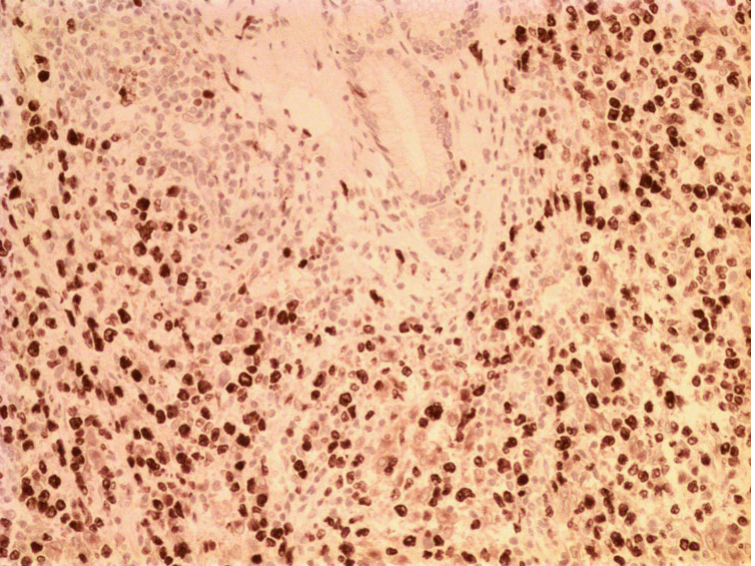
High mitotic activity highlighted by Ki67 expression (Ki67 × 200).

At surgery, a large mass at the pancreatic head was found, which could be easily separated from the contiguous vascular structures with blunt dissection. Interestingly, the tumour did not seem macroscopically to invade the surrounding viscera. A pancreaticoduodenectomy (Whipple procedure) was performed. Histologic evaluation of the surgical specimen revealed extensive involvement of the pancreas by a diffuse, extranodal, high grade, large cell, NHL (centroblastic lymphoma). A neoplastic population, consisting of large non-cleaved lymphoid cells with nucleoli, was seen to surround pancreatic ducts; no lymphoepithelial lesions were present (Figure 2). A few small reactive T lymphocytes CD3+ and CD45R0+ (Dako, Glosrtup, Denmark) were observed. Immunohistochemical assessment of the neoplastic cells revealed B lymphoid phenotypes CD20+ (Dako) (Figure 3) and CD10+ (Cell Marque Corporation, USA), and the cells were negative for CD3, CD45R0, CD30, CD15, ALK (anaplastic lymphoma kinase), EMA (epithelial membrane antigen) (Dako) and EBN-A2 (Epstein-Barr virus nuclear antigen 2) (Dako). Mitotic activity was very high (Ki67 (Dako) > 80%) (Fig. 4). The tumour was classified as stage IIE according to the Ann Arbor classification [8,9]. The resection margins were free of disease. However, all adjacent lymph nodes in the gastric or peripancreatic region were infiltrated by the tumour.

In the immediate postoperative course, the patient had several episodes of bilious vomiting shortly after food intake, attributed to oedema of the gastrojejunostomy, which gradually resolved. He also developed a pancreatic fistula which completely resolved after 5 weeks without affecting the patient's nutritional status or electrolyte balance.

After surgery the patient underwent four chemotherapeutic sessions consisting of cyclophosphamide, novantrone, vincristine and prednisone, all administered at 3-week intervals. Twenty-one months after surgery the patient was alive and in a good clinical condition.

## Discussion

PPL is a very rare neoplasm that may be confused with adenocarcinoma, the most common neoplasm of the pancreas. An extensive review of the international literature has revealed a total of 162 adult patients with biopsy-proven NHL primarily involving the pancreas (Table 1) [2,3,10-17]. The clinical manifestation of PPL is non-specific and differentiation from pancreatic adenocarcinoma on a clinical basis is difficult. Only in cases where a patient presents with abdominal pain and a palpable mass without jaundice is the clinical suspicion of pancreatic lymphoma, over adenocarcinoma, enhanced.

**Table 1:** Literature review of primary pancreatic lymphoma: patients' characteristics, pathologic types, treatment and outcome

First author	Year	Number (*n*)	Pathology	Operation	Chemotherapy	Radiotherapy	Outcome	Response
Feather	1951	1	DLBCL	Total Pancreatectomy	None	None	16 months – NED	CR
Acherman	1976	3	DLBCL	None	CHOP	Yes	12 months – NED	CR
Boddie	1980	2	DLBCL					
Freed	1983	1	NR	Distal Pancreatectomy	None	None	12 months – NED	CR
Teefy	1986	2	DLBCL (1) FLCL (1)	None	Yes	Yes	12 months – Death	PR
Hart	1987	14	Histiocytic (9) Poorly diff lymphocytic (2) Well diff lymphocytic (1) T-cell (1) Undifferentiated(1)	Distal Pancreatectomy (1)	CHOP (14)	1 of 14	NR (1 with resection) Death (7) at 3 to 108 months)	NR
Webb	1988	9	DLBCL (6) Follicular mixed (2) Small cleaved (1)	Distal Pancreatectomy (1)	MACOP-B (8) CAMEL (1)	None	95 months-NED (1) 21 months-NED (8)	CR
Mansour	1989	12	NR	Distal Pancreatectomy(1)	CHOP (11)	5 of 9	Death (5) in 2 to 11 months, Recurrence (2) in 3 and 48 months	PR
Cappell	1989	1	DLBCL					
Tuchek	1993	7	NR	None	CHOP (1) CVP (1) CAMEL (1)	NR	Death (4) Alive (3) at 60, 72, 96 months	CR
Borrowdale	1994	1	NR	Whipple	Yes	None	Death in 12 months	CR
Behrns	1994	12	DLBCL (7) Small cleaved (2) Mixed (3)	Whipple (1) Bypass (4)	CHOP (4)	4 of 12	Death in 38 months at most	PR
Fidias	1995	3	DLBCL	None	CHOP	None	Death (1) Recurrence (1) NED in 36 months(1)	CR (2) PR (1)
Tanaka	1996	1	DLBCL (1)	Whipple	CHOP	NR	NED in 36 months	CR
Ezzat	1996	5	DLBCL (5)	Whipple (1)	CHOP (4) CHOP-Ble (1)	NR	NED in 21 to 84 months (4)	PR
Rumstadt	1997	2	DLBCL (2)	Whipple (2)	CHOP (1)	None	NED in 29 months	CR
Bouvet	1997	11	DLBCL (10) Mixed follicular (1)	Whipple (1) Distal Pancreaticoduo-denectomy (2)	CHOP-Bleo	7of 11	Death (3) Recurrence (2) NED (6)	CR
James	1997	2	DLBCL (1)	None	CHOP (1) PACEBO + CHOP (1)	1 of 2	NED in 24 months	PR
Koniaris	2000	8	DLBCL (7) Nixed follicular (1)	Whipple (3)	CHOP (6) MACOP-B	None	Death (2) NED (3, resection)	CR
Islam	2001	1	DLBCL	Bypass, biopsy	CHOP	None	NED in 18 months	CR
Nishimura	2001	19	DLBCL (15) T-cell (4)	Pancreatectomy(10)	CHOP (5) Mitomycin Tegafur (1) Unspecified chemo (1)	NR	1-year actuarial survival B-cell: 51.9% T-cell: 0%	PR
Boni	2002	1	DLBCL (1)	Laparoscopy-biopsy	Chemo (1)	None	NED in 9 months	CR
Hauksson	2002	1	DLBCL	PTC stenting biopsy	Chemo	NR	NR	NR
Volmar	2004	14	DLBCL (6) follicular (4) Suggestive of lymphoma (3) B lymphoma (1)	No	Yes	NR	Death (3) Alive (11) in a mean 11.8- month followup	2 PR 11 CR
Nayer	2004^10^	8	DLBCL (4) High-grade B-cell (1) Low grade B (2) Suspicious (1)	None	Chemo (4) Chemo and auto SCT (2) Nr (2)	3 of 8	6 alive at 2 to 6 months	PR
Pezzilli	2004	1	T-cell lymphoma	None	Chemo (1)	None	Death (1)	NR
Arcari	2005	5	DLBCL (3) Lymphoplasmacytic (2)	Pancreatectomy (2)	CVP/CHOP(3) CHOP(1) CVP(1)	2 of 5	Death (3) Alive 2, (1, resection)	PR
Grimison	2005	4	DLBCL (3) Follicular (1)	None	CHOP(2) Rituximab-CHOP(1) CVP(1)	4	Death 132 months Alive 3 at 15, 25, 64 months	PR
Ji	2005	1	DLBCL (1)	Whipple (1)	NR	NR	NR	NR
Savopoulos	2005	1	ALCL (1)	Biopsy	None	None	Died 2^nd ^postoperative day	-
Kang	2006	1	Non-Hodgkin's lymphoma	Biopsy	NR	NR	NR	NR
Lin	2006	6	DLBCL (6)	Whipple (2) Pancreatectomy (1) Biliary decompression (1)	Chemo (6)	γ-radiotherapy (1)	Death (3) at 2, 37, 49 months NED in 21 months (1)	PR
Battula	2006	1	DLBCL (1)	Whipple (1)	CHOP (1)	NR	NR	NR
Liakakos	2007	1	DLBCL (1)	Whipple (1)	Cyclophosphamide Novantrone Vincristine Prednisone	No	Alive (2 years)	PR

DLBCL, diffuse large B-cell lymphoma; FLCL, follicular large cell lymphoma; CHOP, cyclophosphamide, doxorubicin, vincristine, prednisolone; PACEBO, prednisolone, hydroxydaunorubicine, cyclophosphamide, etoposide, bleomycin, vincristine; CAMEL, cyclophosphamide, adriamycin, vincristine, prednisone; CVP, cyclophosphamide, vincristine, prednisone; SCT, stem cell transplantation; NED, no evidence of disease; NR, not reported; CR, complete response; PR, partial response

There are some biochemical markers which, in conjunction with suspicious clinical manifestations, may help focus the physician's attention on the possibility of a pancreatic lymphoma. More specifically, lactate dehydrogenase (LDH) and β2 microglobulin are considered to be tumour markers in lymphoproliferative disorders and have an important positive prognostic value. Serum carbohydrate antigen 19-9 (CA 19-9) level in patients with PPL is usually not elevated. This is in contrast with pancreatic adenocarcinoma, in which almost 80% of cases have a high CA19-9 level [11].

Pancreatic lymphoma, like most extralymphatic lymphomas, is predominantly of intermediate or high-grade histology, with diffuse large B-cell lymphoma being the predominant type [12]. Less than 20% of reported cases demonstrate low-grade histology. The majority of pancreatic lymphomas reported to date in the literature have been classified as of B-cell type, but several cases of T-cell lymphomas have also been described [13].

CT is the most common imaging technique used in the detection and characterization of PPL. On CT, two different morphologic patterns of pancreatic involvement are seen; one is a localized, well-circumscribed tumour with diffuse enlargement infiltrating most of the pancreatic gland. This pattern may mimic the imaging findings of acute pancreatitis with gland enlargement and irregular infiltration of the peripancreatic fat [14]. The well-circumscribed tumoural form can be easily misinterpreted as a ductal adenocarcinoma, especially in patients with dilatation or encasement of the pancreatic and common bile ducts [15]. In contrast, the combination of a bulky, localized tumour in the pancreatic head without significant dilatation of the main pancreatic duct, as seen in our case, strengthens a diagnosis of pancreatic lymphoma over adenocarcinoma. Furthermore, if enlarged lymph nodes are encountered below the level of the renal veins, in association with a large pancreatic tumour, virtual exclusion of adenocarcinoma is possible [14].

Contrast-enhanced CT also offers certain criteria to define the resectability of pancreatic tumours: the absence of extrapancreatic disease; preservation of the fat plane between the tumour and the confluence of superior mesenteric and portal vein (SMPV); and absence of tumour involving or encasing the SMA, coeliac or hepatic arteries [15]. Therefore, one may rely on the CT findings without the need for additional imaging studies in order to evaluate the resectability of a pancreatic head tumour.

Although certain serum abnormalities and CT changes are suggestive of lymphoma, tissue examination is essential for diagnosis. Patients presenting with advanced disease may be diagnosed by peripheral lymph node FNA, core or open biopsy [2,16]. However, there are cases in which the performance of explorative laparotomy becomes mandatory due to the inability of the aforementioned diagnostic modalities to differentiate between pancreatic adenocarcinoma and some other types of pancreatic malignancy [4,11].

At present, the standard protocols for management of pancreatic lymphomas include a number of chemotherapy regimens [10]. The most commonly used regimen is the first-generation therapy CHOP [2], but a range of second- and third-generation regimens are also used. Expected outcomes for all NHL treatments, based on multi-institutional studies using CHOP or an equivalent chemotherapy, with or without radiotherapy, regardless of location, are a complete response rate of approximately 50%, and a partial response rate of approximately 30%. Overall, a 3-year disease-free survival rate of 46% has been reported after such therapies [6].

The observed moderate long-term survival rates for pancreatic NHL with chemotherapy and radiotherapy alone, and the improvements in morbidity and mortality associated with pancreatic surgery, call for a re-evaluation of therapeutic strategies for NHL of the pancreas [17]. A retrospective analysis and a comparison of 15 surgically treated patients with PPL (treated between 1951 and 2000 in several centres) with non-operatively treated stage I or II pancreatic NHL patients (encountered over a similar period) by Koniaris et al. [2] demonstrated markedly improved complete remission and cure rates in the surgically treated group. Detailed comparison between the surgically treated and non-surgically treated groups reveals that age, histologic subtypes and male to female ratio were similar in the two groups. Tumour size was clearly larger in the non-operated group and in many cases patients treated conservatively had unresectable disease. However, the successfully resected group represented a dramatic complete response rate of 100% and long-term survival rate of 94%. These data suggest that for surgically resectable stage I or II pancreatic NHL, resection should be an option in the multimodal therapeutic regimen [11]. To the potential role of surgery in the treatment of pancreatic NHL should be added its well-established place in the management of lymphoma involving other GI organ systems, such as gastric and small bowel lymphomas [7].

## Conclusion

When a patient with a pancreatic mass is encountered, preoperative contrast-enhanced CT or magnetic resonance imaging should be obtained. Only in cases in which the resectability of a pancreatic head mass is regarded as impossible, according to the above criteria, should the performance of a definite tissue diagnosis be contemplated. This is achieved with the aid of percutaneous FNA and flow cytometry analysis. If a mass located in the head of the pancreas is regarded as potentially resectable, we advocate explorative laparotomy of the patient. If the intraoperative findings confirm the feasibility of resection of the tumour, pancreaticoduodenectomy is advocated, provided that the surgery is performed by surgeons well-practised with the nuances required for safe pancreatic resection, especially in the case of PPLs, which also respond well to treatment with chemotherapy and radiotherapy alone.

## Abbreviations

ALT: alanine transaminase; AST: aspartate transaminase; CA 19-9: carbohydrate antigen 19-9; CHOP: cyclophosphamide, doxorubicin, vincristine and dexamethazone; CT: computed tomography; EBN-A2: Epstein-Barr virus nuclear antigen 2; EMA: epithelial membrane antigen; FNA: fine needle aspiration; GGT: Gamma glutamyl transpeptidase; GI: gastrointestinal; LDH: lactate dehydrogenase; NHL: non-Hodgkin's lymphomas; PPL: primary pancreatic lymphoma; SMA: superior mesenteric artery; SMPV: superior mesenteric and portal vein; SMV: superior mesenteric vein.

## Competing interests

The authors declare that they have no competing interests.

## Authors' contributions

TL and GK participated in this patient's medical and surgical management. IN was the pathologist who examined the surgical specimen and produced all histological photos in this paper. EPM and DT participated in the acquisition of data and wrote the manuscript. All authors read and approved the final manuscript.

## Consent

Written informed consent was obtained from the patient for publication of this case report and accompanying images. A copy of the written consent is available for review by the Editor-in-Chief of this journal.
